# Immunization with mRNA-LNP Elicits De Novo IgG Responses in the Presence of Maternal Antibody

**DOI:** 10.3390/vaccines14090769

**Published:** 2026-09-02

**Authors:** John M. Ramos, Brittany Plummer, Christian R. Binuya, Mackensie Gross, Adelaide S. Fuller, Krithika P. Karthigeyan, Savannah Berrios, Sallie R. Permar, Caitlin A. Williams

**Affiliations:** Division of Infectious Disease, Department of Pediatrics, Weill Cornell Medical College, New York, NY 10021, USAsap4017@med.cornell.edu (S.R.P.)

**Keywords:** SARS-CoV-2, maternal antibody, early-life immunization, neonatal immunity

## Abstract

Background/Objectives: Maternal antibodies can inhibit vaccine-specific humoral responses in early life, leaving infants at increased risk for severe disease for vaccine-preventable infections. In the case of SARS-CoV-2, infants under the age of 3 months represented most child hospitalizations, yet there is no approved vaccine for children under the age of 6 months. There is a clear need for effective immunization in early life to prevent infant morbidity and mortality. Here, we established a mouse model to define how maternally derived antibodies shape early-life responses to mRNA vaccination. Methods: Adult female mice were immunized with PBS or 5 mcg of the SARS-CoV-2 mRNA-1273 vaccine via intramuscular injection and paired with a male. Pups from subsequent litters were immunized with PBS or 5 mcg of mRNA-1273 vaccine via intramuscular injection. Peripheral blood and spleens were collected at time points post-immunization. We measured vaccine-elicited anti-Spike IgG in mouse pups exposed or unexposed to vaccine-specific maternal IgG. Results: Spike-specific maternal IgG is detectable at high levels immediately after pup immunization or mock immunization; however, in mock immunized pups, it wanes by three weeks post-pup immunization. Pups born to immunized dams developed Spike specific IgG comparable to pups born to naïve dams. IgG subclass analyses distinguished passively acquired antibodies from vaccine-induced responses. Despite robust binding antibody responses, neutralizing activity against D614G pseudovirus was heterogeneous and did not scale proportionally with IgG titers, showing qualitative differences in early-life humoral immunity. Splenic Spike-specific B cell frequencies and T follicular helper (Tfh) cell responses were detectable in vaccinated pups irrespective of maternal immunization status, with Tfh cell frequencies peaking at day 7 post-immunization in both groups. Conclusions: Using SARS-CoV-2 as a model pathogen, we found that early-life mRNA vaccination can elicit humoral immune responses in the presence of maternal antibodies. Furthermore, the presence of maternal antibody did not inhibit the development of antigen-specific B cells or Tfh cells in the spleen. Our findings support the potential of extending vaccination strategies into early infancy and provide a framework for optimizing mRNA-based vaccine timing and design in the context of maternal immunity.

## 1. Introduction

Maternal antibodies play a crucial role in mitigating infectious disease in early life. Infants born to immunized mothers are less likely to be hospitalized due to viral infections such as influenza, SARS-CoV-2, and respiratory syncytial virus (RSV) [1,2,3,4]. Yet, maternal antibodies can also inhibit infant vaccine-elicited immune responses through mechanisms such as epitope masking or by engaging the inhibitory Fcγ receptor IIB (FcγRIIB) [5,6]. This effect is found in protein subunit vaccines such as acellular pertussis but is most pronounced for live attenuated vaccines such as the measles vaccine, where even low levels of maternal antibodies diminish the vaccine efficacy, significantly influencing vaccination schedules globally [7,8,9,10].

To reduce concerns about interference from maternally derived antibodies, many infant immunization strategies have historically deferred vaccination until later in infancy, when these antibodies are presumed to have waned. This framework was applied to the mRNA–LNP SARS-CoV-2 vaccines, for which vaccination is currently limited to infants aged 6 months or older. While this age-based threshold simplifies concerns regarding maternal antibody interference, it leaves a substantial period of early infancy without direct vaccine-induced protection [11]. During this period, infants younger than 6 months experienced the highest COVID-19 hospitalization rates among pediatric age groups, highlighting the vulnerability of this population and the need to evaluate immunization strategies earlier in life [12,13,14]. Specifically, from October 2023 to April 2024, COVID-19 hospitalization rates among young infants were higher than rates among all other age groups except adults aged ≥75 years and were comparable to rates in adults aged 65–74 years [14].

Infants and young children immunized with the Moderna (mRNA-1273) or Pfizer (BNT162b2) mRNA–LNP vaccines achieved immune responses comparable to those observed in older children, but only through distinct dose and schedule regimens, including a three-dose low-dose series for BNT162b2 and a two-dose higher-dose series for mRNA-1273 [12,13]. Our group previously reported that the age at immunization did not significantly impact immune responses approximately 1 month after primary series completion in children under 5 years, including in infants under 12 months who had measurable levels of maternally derived antibodies [14]. Maternal antibody interference with vaccine-elicited immunity has been documented across multiple model systems, including murine models of live-attenuated vaccines and neonatal non-human primate HIV immunization studies [9,15,16,17] with varying degrees of interference, yet the impact of maternal antibody on mRNA-LNP vaccine responses in early life remains incompletely characterized [18,19].

To further investigate the potential impact of maternal antibody on the mRNA-LNP-elicited immune responses, we developed a murine model to study the effect of pre-existing maternal antibody responses against SARS-CoV-2 on infant SARS-CoV-2 mRNA-LNP vaccine-elicited humoral immunity. Unlike humans, rodents transfer breastmilk-derived antibody from the intestinal lumen into the pup peripheral blood mediated by the neonatal Fc receptor (FcRn), which functions analogously to transplacental transfer of maternal antibody in humans [20,21,22]. This mechanism enables an analysis of the impact of high-titer vaccine-elicited maternal antibody in weanling pups. Previous studies of murine maternal immunization using a protein subunit vaccine have demonstrated that intramuscular vaccine administration elicits high titers of vaccine-specific IgG in mouse milk, with subsequent transfer of maternal IgG to pups [23]. Here, we utilized the murine model to examine maternal antibody at two immunological stages: the neonatal stage wherein maternal antibody is actively increasing over the study period and the weanling stage when maternal antibody is at its peak and wanes over the study period. We employed four methods of antibody analysis: total anti-Spike IgG levels in pups, using crossbreeding to measure pup derived vs. maternally acquired anti-Spike IgG levels in pup sera, serum neutralization of SARS-CoV-2 pseudovirus, and Spike-specific B cell comparisons. We demonstrate that immunized pups from immunized dams can develop de novo Spike IgG responses that are comparable to those in pups from naïve dams, suggesting that the mRNA-LNP platform may be able to overcome maternal antibody inhibition and support vaccination prior to maternal antibody waning.

## 2. Materials and Methods

### 2.1. Animal Study Design

**Figure 1:**
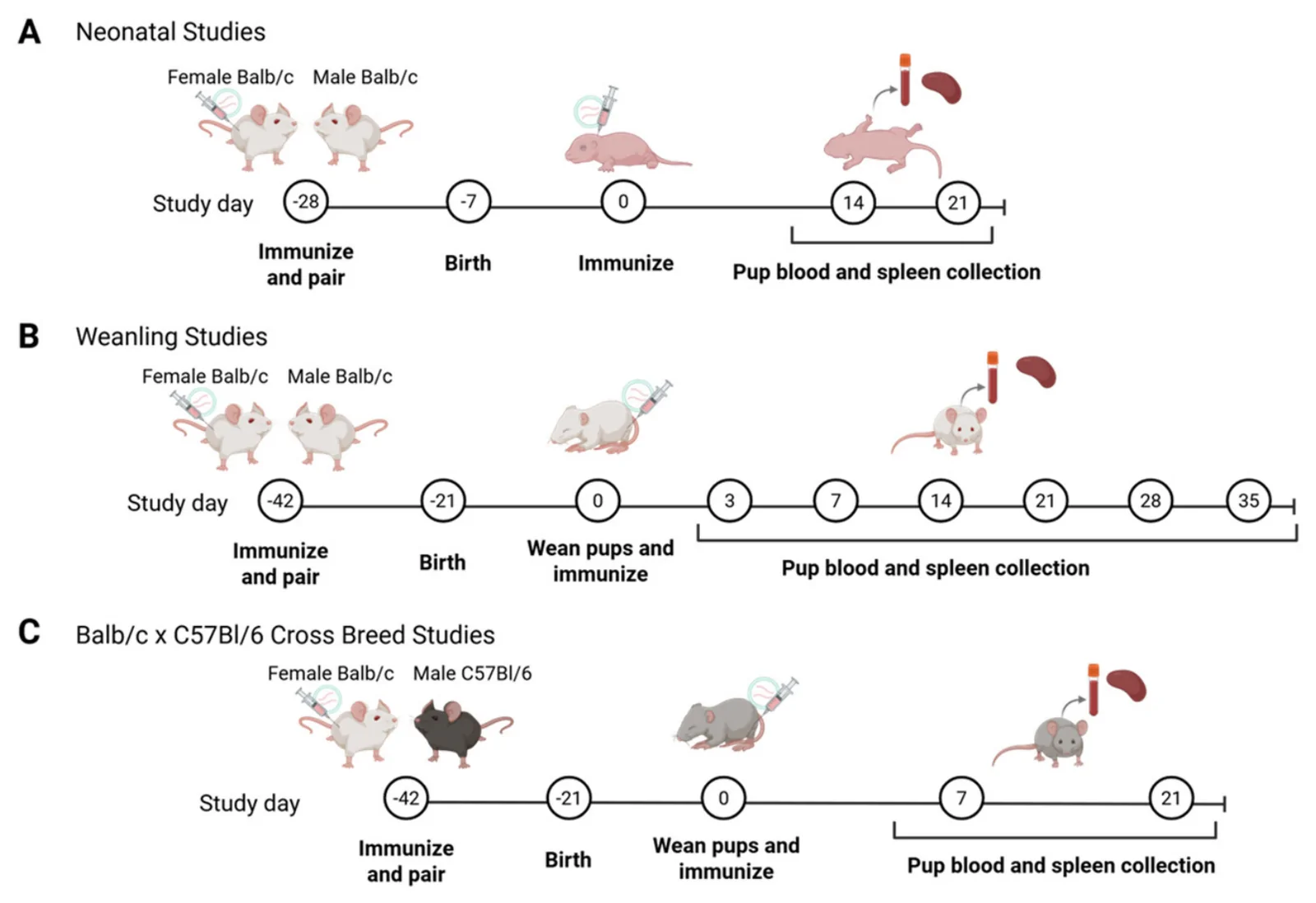
Experimental timeline for SARS-CoV-2 maternal–fetal immune transfer. (**A**) Neonatal studies: BALB/c dams were bred with BALB/c males and immunized on day –28 via intramuscular injection. Pups were immunized on day 7 of life by intradermal injection of 4 mcg of mRNA-1273. (**B**) Weanling studies: BALB/c dams were bred with BALB/c males and immunized on day –42 via intramuscular injection. Pups were immunized with 5 mcg of mRNA-1273. (**C**) Crossbreed studies: BALB/c dams were bred with C57BL/6 males. Pups were immunized with 5 mcg of mRNA-1273. In all studies, dams were immunized with either PBS or 5 mcg of mRNA-1273. Peripheral blood and spleens were collected at the time points indicated. Figure made in BioRender Created in BioRender. Permar, S. https://BioRender.com/61jw1y9 (accessed on 23 August 2026).

BALB/c, an inbred strain of mice used for general purposes which expresses IgG2a, (Taconic, Rensselaer, NY, USA) dams aged 6–8 weeks, were immunized via intramuscular (IM) injection with either PBS or mRNA-1273 (Moderna COVID-19 vaccine) at the time of breeding. To produce approximately five litters per study group, two to three females were paired with a single male. Vaginal plugs were checked 18–24 h after pairing. Males were removed after one week of co-housing. Females were subsequently housed individually beginning one week prior to expected parturition. For the crossbreed studies, BALB/c dams were bred with C57BL/6 males, a common-use inbred mouse strain which expresses IgG2c, (Taconic, NY, USA). All pups were immunized on study day 0. For the neonatal study group, pups were immunized on day 7 of life by intradermal injection of either PBS of 4 mcg of mRNA-1273, and blood and spleens were collected on study days 14 and 21 (Figure 1). For the weanling and crossbreed study groups, pups were immunized at weaning by IM injection with either PBS (maternal vaccine only group) or 5 mcg of mRNA-1273, and blood was collected at 0, 3, 7, 14, 28, and 35 days post-pup immunization for plasma processing (Figure 1, Table 1). A subset of pups from each group were euthanized for spleen collection and subsequent B cell analysis (Table 1). Due to limited availability of mRNA-LNP vaccines following their initial Emergency Use Authorization, weanling and neonatal pup studies used Moderna mRNA-1273 multidose vials (red-cap) obtained from the Weill Cornell Medical College Vaccine Clinic. Vials were thawed but unopened and were used within one year of the labeled beyond-use date. For crossbreed studies, mice were immunized with commercially sourced, Moderna mRNA-1273 vaccine (McKesson, Irving, TX, USA). Adult and weanling mice received 50 µL IM injections of 5 mcg mRNA in the hind limb. Vaccine doses were diluted with sterile PBS to achieve a concentration of 5 mcg per 50 µL. Neonatal pups received 10 µL intradermal (ID) injections of 4 mcg mRNA into the pinna of each ear following brief anesthesia by placement on ice; pups were then warmed and monitored until fully conscious before being returned to the dam. Vaccine doses were diluted to 4 mcg per 20 µL. All animal studies and procedures were approved by the Institutional Animal Care and Use Committee (IACUC) of Weill Cornell Medicine (protocol: 2022-0007).

Abbreviations: AF, Alexa Fluor; BB, BD Horizon BB; BV, Brilliant Violet; PE, phycoerythrin; SB, Super Bright; BP, band pass; Conc., concentration. SARS-CoV-2 Spike protein (S1+S2 ECD, D614G, His-tagged; Sino Biological #40589-V08B4 Paoli, PA, USA) was biotinylated in-house using BirA ligase and coupled to fluorescent streptavidin conjugates for antigen-specific B cell detection.

### 2.2. Blood Collection

Blood from weanling pups at the 3-week time point was collected by cardiac puncture following euthanasia by carbon dioxide asphyxiation and cervical dislocation. For mice older than 3 weeks, blood was obtained by tail-vein nick or submandibular venipuncture. Neonatal pups were euthanized by decapitation, and blood was collected from the trunk. All samples were collected using capillary blood collection tubes (Sarstedt KMIC-SER, Sarstedt, Nümbrecht, Germany).

### 2.3. IgG-Binding Assays

IgG binding to the SARS-CoV-2 D614G Spike protein (40589-V08B4, Sino Biological, Paoli, PA, USA) was measured by enzyme-linked immunosorbent assay (ELISA). High-binding 384-well plates (Corning Glendale, AZ, USA) were coated overnight at 4 °C with Spike protein at 2 mcg/mL in 0.1 M sodium bicarbonate. Plates were washed (14.5% 25X PBS, 3.5% Tween-20) using an automated plate washer (BioTek, Agilent, Santa Clara, CA, USA) and blocked for 1 h with blocking solution (PBS, 4% whey protein, 15% goat serum, 0.5% Tween-20). Serum samples were serially diluted to generate an 8-point two-fold serial dilution starting at 1:100 and incubated for one hour at room temperature. After washing twice, the plates were incubated for one hour with horseradish peroxidase-conjugated anti-mouse IgG (Southern Biotech, Birmingham, AL, USA, 0107-05) diluted 1:5000 in blocking solution. SureBlue Reserve TMB Microwell Peroxidase Substrate (Cat. No. 50-674-97 KPL, Fisher Scientific, Pittsburgh, PA, USA) was added to the wells for 3 minutes and stopped with 1% HCl (Cat. No. 50-674-44 KPL, Fisher Scientific, Pittsburgh, PA, USA). Plates were read at 450 nm. The area under the curve (AUC) and ED_50_, four-parameter logistic regression, were determined using GraphPad Prism (version 10).

### 2.4. IgG Subclass

IgG subclass binding was assessed by ELISA as described in Section 2.3, with subclass-specific detection performed using subclass-specific monoclonal antibodies. IgG2a antibodies were detected using HRP-conjugated Goat Anti-Mouse IgG2a (Cat. No. 1080-05Southern Biotech, Birmingham, AL, USA). IgG2c antibodies were detected using HRP-conjugated Goat Anti-Mouse IgG2c Human ads (Cat. No. 1079-05Southern Biotech, Birmingham, AL. USA). All secondary antibodies were diluted 1:2000.

### 2.5. Pseudoviruses and Cell Lines

Pseudovirus production was performed as previously described [14]. Briefly, plasmids encoding the SARS-CoV-2 full-length Spike protein, a CMV luciferase reporter, and a CMV promoter were co-transfected into HEK293T cells (ATCC, Manassas, VA, USA) using Fugene HD transfection reagent (Promega, Madison, WI, USA). Cell culture supernatant was collected and clarified by low-speed centrifugation (300× *g*) for 10 min to remove cellular debris. Pseudovirus titer, expressed as the tissue culture infectious dose at 50% (TCID_50_), was determined by serial dilution and incubation with HEK293T cells expressing human ACE2 and TMPRSS2 (BEI Resources) in DMEM supplemented with 10% FBS and Penicillin-Streptomycin. After 72 h, the cell supernatant was removed; cells were lysed in 1× lysis buffer (Cat. No. E1531 Promega, Madison, WI, USA), and Bright-Glo luciferase reagent was added. Luminescence was measured using the BioTek Synergy plate reader. Cells used for pseudovirus production, titration, and neutralization were maintained in DMEM supplemented with 10% FBS.

### 2.6. SARS-CoV-2 Pseudovirus Neutralization Assays

Neutralizing activity was assessed using D614G SARS-CoV-2 Spike-pseudotyped lentiviral particles, quantified by reduction in firefly luciferase reporter activity. Serum samples were initially diluted 1:20 in assay medium (89% DMEM, 10% heat-inactivated FBS, 1% Penicillin-Streptomycin), then serially diluted three-fold in duplicate (1:20 to 1:1,562,500), and incubated with pseudovirus for 45–90 min at 37 °C in poly-L-lysine-coated 96-well plates (Cat. No. 3841Corning, Glendale, AZ, USA). HEK293T cells (10,000 cells per well in 100 µL) were then added immediately to each well. After 72 h, cells were lysed in 1× Promega lysis buffer, incubated with Bright-Glo luciferase reagent, and luminescence was measured on black-bottom 96-well plates (Corning) using a BioTek Synergy plate reader. The 50% inhibitory dilution (ID50) was calculated from the resulting neutralization curves using four-parameter logistic regression in GraphPad Prism (version 10). Samples with an ID50 below the lowest serum dilution tested (1:20) were assigned a value of 20.

### 2.7. Splenocyte Isolation

Spleens were harvested and mechanically dissociated through a 70 µm cell strainer (Cat. No. 352350). Corning, Glendale, AZ, USA Red blood cells were lysed using ACK lysing buffer (Cat. No. A10492-01,ThermoFisher, Waltham, Massachusetts, USA). The cell suspension was layered onto 5 mL Lympholyte-M (Cat. No. CL5120, Cedarlane, Burlington, ON, CAN ) and centrifuged at 2600 rpm for 25 min at 23 °C. Purified splenocytes were cryopreserved in liquid nitrogen until analysis.

### 2.8. Identification of Spike-Specific B Cells and T Follicular Helper Cells by Flow Cytometry

Cryopreserved splenocytes (1 × 10^6^ cells per sample) were thawed and stained for surface and intracellular markers prior to acquisition on a Cytek Aurora spectral flow cytometer (Cytek Biosciences, Fremont, CA, USA). Live/dead staining was performed using LIVE/DEAD Lime (506) viability dye (1:1000 dilution, 100 µL per well) to exclude non-viable cells from analysis. Surface staining was performed with 50 µL of antibody master mixture containing CD20, CXCR5, CD4, PD1, and dual-labeled SARS-CoV-2 Spike protein (AF488 and BV421) for 20 min at room temperature (Table 2). Cells were fixed in 100 µL of 4% paraformaldehyde for 15 min, then permeabilized. Cells were then washed with phosphate-buffered saline with fetal bovine serum (PBSF) and incubated with 50 µL of Ki67 and BCL6 antibody mixture for 20 min at room temperature. Cells were washed with PBSF and stored at 4 °C, protected from light, until acquisition.

Single-color controls were prepared using compensation beads. Viability-dye compensation controls were prepared using one drop of Amine Reactive Compensation (ARC) reactive beads incubated with 3 µL of viability dye for 30 min, protected from light. After washing in 2 mL PBSF and centrifuging at 300× *g* for 5 min, beads were resuspended in 400 µL PBSF and combined with one drop of ARC-negative beads. All samples and controls were acquired on a Cytek Aurora using manufacturer-recommended settings and analyzed using FlowJo 10 software. Representative gating strategies are shown in Supplementary Figure S1. 

### 2.9. Statistical Analysis

All statistical analyses were performed using GraphPad Prism (version 10). Antibody binding titers (ED_50_) and neutralization titers (ID_50_) were log10-transformed prior to analysis. Group comparisons at individual time points were performed using two-way analysis of variance (ANOVA). All data and statistical analysis will be made available through GitHub at https://github.com/PermarLab (accessed on 23 August 2026).

## 3. Results

### 3.1. mRNA-LNP Vaccines Are Only Mildly Immunogenic in Neonatal Pups

**Figure 2:**
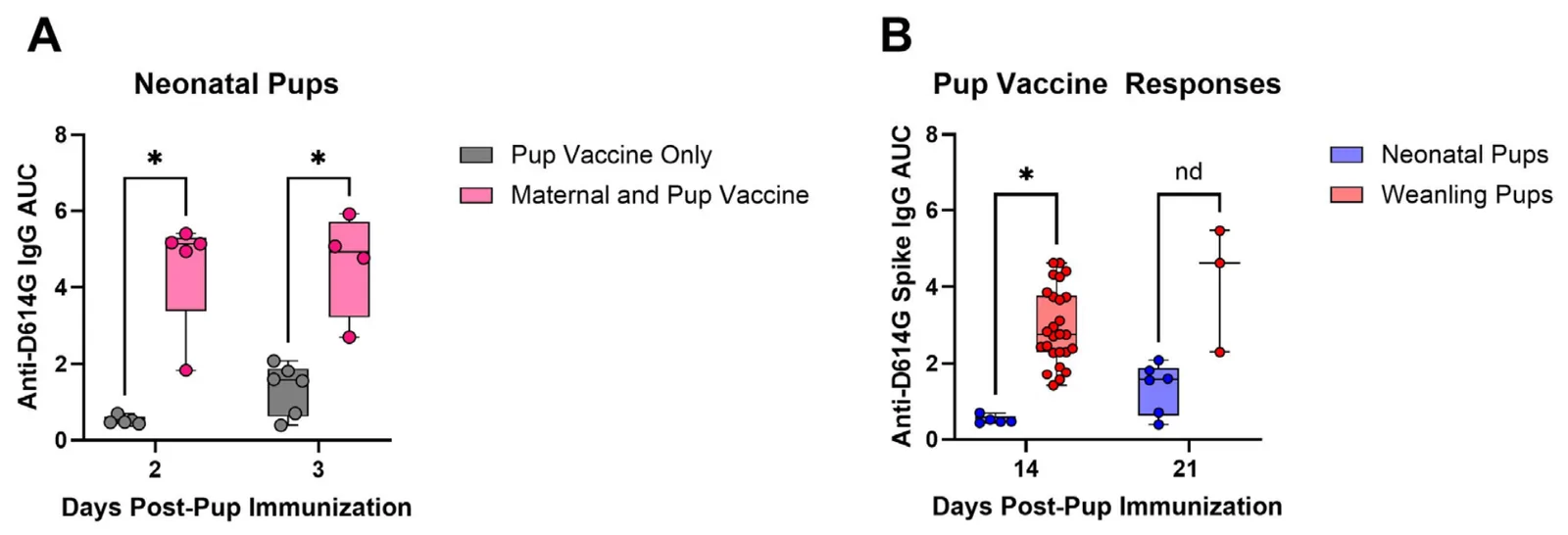
Vaccine-elicited anti-D614G Spike IgG responses in neonatal mice. Anti-Spike IgG was measured by ELISA by incubating serum samples diluted 1:100 in Spike-coated wells for 1 h at room temperature followed by detection with anti-mouse IgG (HRP) secondary antibody. (**A**) Area under the curve (AUC) of anti-Spike IgG in neonatal pup serum. Pups were immunized on day 7 of life via intradermal injection of 4 mcg of mRNA-1273. Each dot represents an individual pup; box and whiskers represent the minimum and maximum. (**B**) Comparison of anti-Spike IgG responses on days 14 and 21 post-vaccination in neonatal pups versus weanling pups. All samples were run in duplicate, and all experiments were conducted using identical assay conditions. The mean of each duplicate point was utilized to calculate the AUC using Graphpad Prism software (version 10). * = *p* < 0.05.

We first developed a neonatal immunization model to determine if mRNA-LNP vaccines can elicit de novo IgG production in the presence of increasing levels of vaccine-specific maternal antibodies. We immunized adult female BALB/c mice intramuscularly with 5 mcg of mRNA-1273 or PBS at the time of pairing with males. Neonatal pups were immunized with 4 mcg of mRNA-1273 by intradermal injection into the pinna of the ear on day 7 of life. SARS-CoV-2 D614G Spike-specific IgG levels were measured at 14 and 21 days post-pup immunization (Figure 1A, Table 1). Spike-specific anti-D614G IgG was detectable at weeks 2 and 3 post-immunization in both groups; however, pups not exposed to maternal antibodies developed only low levels of anti-Spike IgG (Figure 2A). As expected, anti-Spike IgG (area under the curve; AUC) levels were significantly higher in pups born to immune dams (median AUC = 5.138, *p* = 0.007) compared to pups born to naïve dams. To determine if these antibody kinetics are age-related, we compared anti-Spike IgG levels to weanlings at the same time points post-pup immunization (Figure 1B, Table 1). We found that neonatal pups developed significantly less anti-Spike IgG than weanling pups (Figure 2B). Thus, we further utilized the weanling pup model for the comparisons of pup vaccine-elicited immune responses in the setting of maternal antibody or no maternal antibody against SARS-CoV-2.

### 3.2. SARS-CoV-2 Spike mRNA-LNP Vaccination Elicits Anti-Spike IgG in Weanling Pups Irrespective of Maternal Immunization Status 

**Figure 3:**
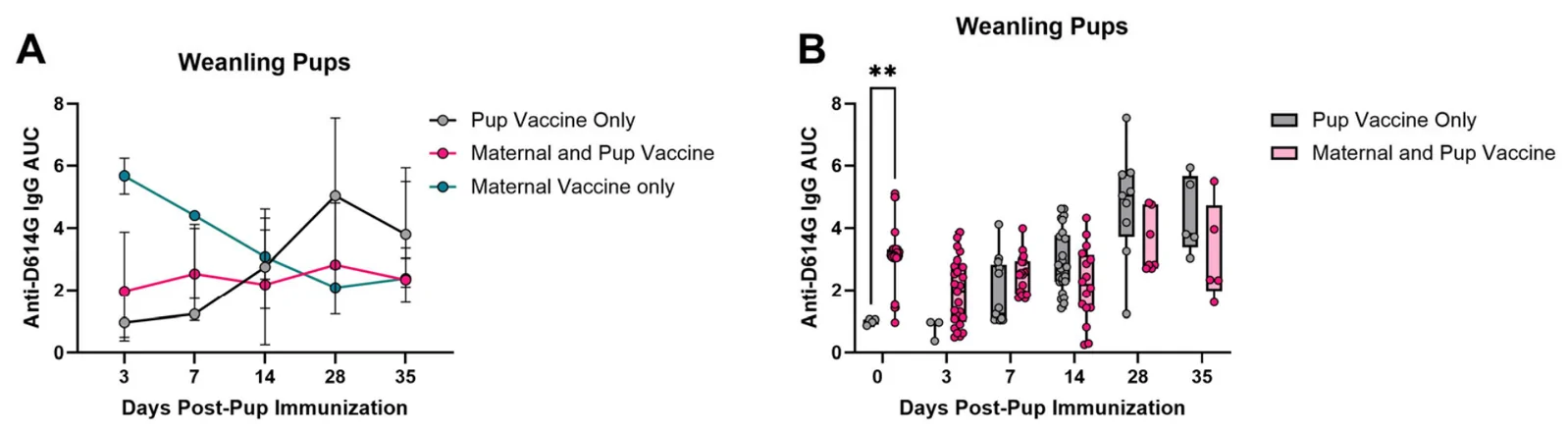
Impact of maternal antibody on vaccine-elicited pup serum IgG levels. Anti-Spike IgG was measured by ELISA in pup sera at regular intervals post-pup immunization. Anti-Spike IgG was measured by incubating serum samples diluted 1:100 in Spike-coated wells for 1 h at room temperature followed by detection with anti-mouse IgG (HRP) secondary antibody. (**A**) shows the area under the curve (AUC) of Anti-D614G IgG detected in the serum of pups born to naïve dams, immunized dams, or mock immunized pups born to immunized dams. The data points represent the median and the range of 4–5 pups for each time point. (**B**) shows the anti-D614G IgG AUC levels in pups born to naïve versus immunized dams. The box and whiskers represent the maximum and minimum, with each data point representing an individual pup. All samples were run in duplicate, and all experiments were conducted using identical assay conditions. The mean of each duplicate point was utilized to calculate the AUC. Statistical comparison was conducted using a two-way ANOVA in GraphPad Prism (version 10). ** = *p* < 0.01.

The SARS-CoV-2 Spike mRNA-LNP vaccine was immunogenic in weanling pups across all vaccination groups (Figure 3). In pups born to naïve dams, anti-Spike IgG levels peaked at day 28 (median AUC = 5.053) (Figure 3A). We measured a median anti-Spike IgG of AUC = 3.124 in pups born to immunized dams. We detected an initial decrease in anti-Spike IgG levels on day 3 (median AUC = 1.9); however, levels of anti-Spike IgG increased by day 7 and remained consistent for the remainder of the study (Figure 3B). Levels of plasma anti-Spike IgG were not statistically significant between pups born to naïve or immune dams, indicating that vaccine-elicited de novo pup IgG was elicited at a comparable level in both groups despite the presence of pre-existing maternal antibody.

### 3.3. IgG Subclass Analysis in Crossbreed Pups Demonstrates Pup-Derived Vaccine Responses in the Presence of Maternal Antibody

To distinguish vaccine-elicited pup antibody from passively acquired maternal IgG in serum, we utilized a model that leverages IgG subclass differences between inbred mouse strains. BALB/c mice predominantly express the IgG2a subclass, whereas C57BL/6 mice express IgG2c [24,25]. Hybrid offspring generated from BALB/c dams crossed with C57BL/6 express both subclasses; as all maternal antibody is derived from the BALB/c dam, anti-Spike IgG2c in these pups serves as an unambiguous, strain-specific readout of de novo pup-derived humoral immunity. BALB/c dams were immunized with either PBS or 5 mcg of mRNA-1273 prior to pairing with C57BL/6 males. Hybrid pups were immunized at 21 days of life, and samples were collected at days 0, 7, and 21 post-pup immunization (Figure 1C, Table 1).

**Figure 4:**
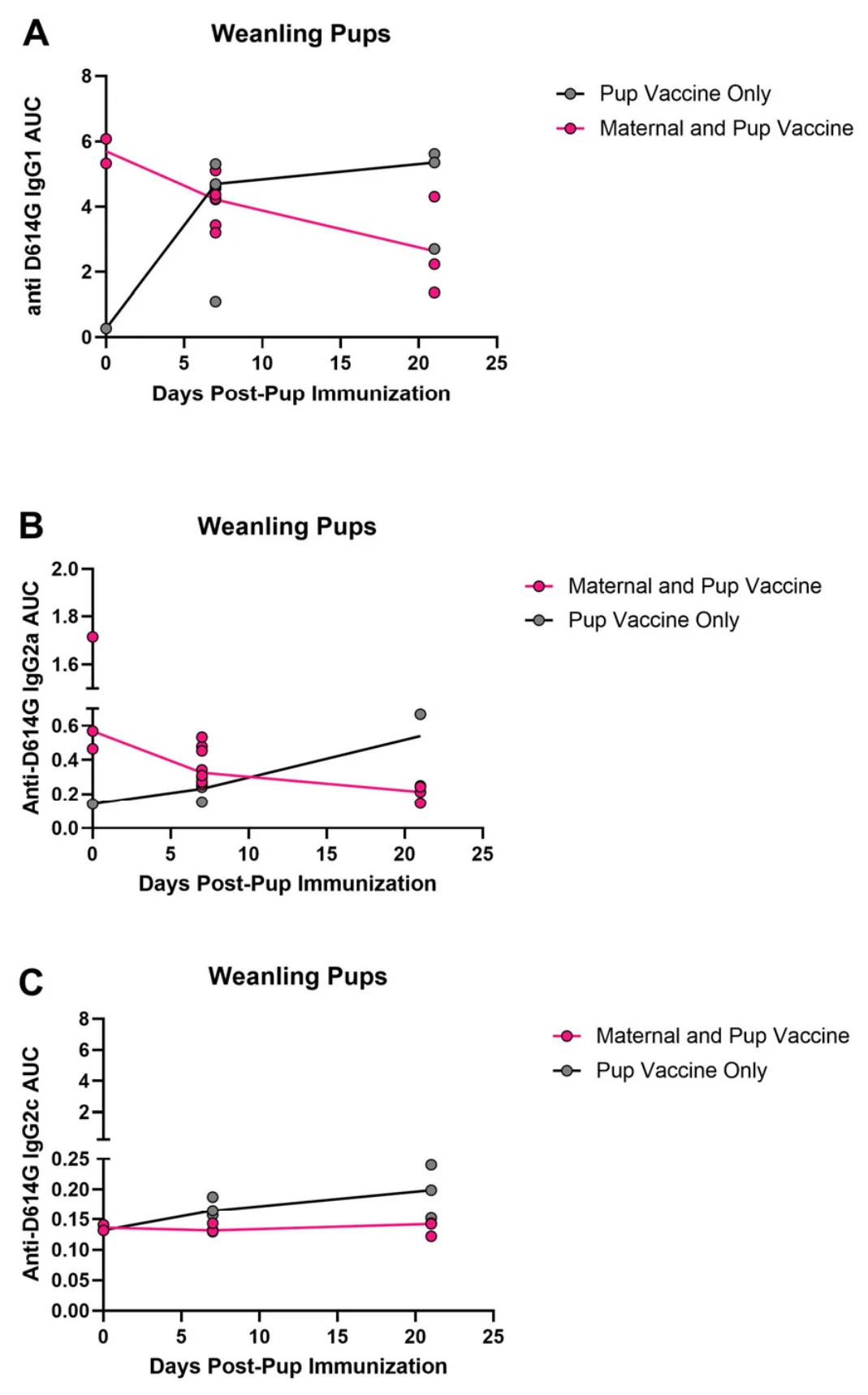
Differentiation of vaccine-elicited Spike-specific IgG from maternal anti-Spike IgG in pup plasma. Anti-Spike IgG was measured by incubating serum samples diluted 1:100 in Spike-coated wells for 1 h at room temperature followed by detection with anti-mouse IgG1, 2a, or 2c (HRP) secondary antibody. (**A**) shows the area under the curve of anti-Spike IgG1 in immunized pups born to immunized or naïve dams. (**B**) shows the area under the curve of anti-Spike IgG2a (Balb/c inherited, dam-derived). (**C**) shows the area under the curve of anti-Spike IgG2c (C57Bl/6 inherited, pup-derived). Each data point represents an individual pup; lines indicate the median. All samples were run in duplicate, and all experiments were conducted using identical assay conditions. The mean of each duplicate point was utilized to calculate the AUC in Graphpad Prism (version 10).

Total anti-Spike IgG1 levels, reflective of vaccine-elicited IgG responses in both the pup and dam, were detectable in the vaccinated pups born to vaccinated dams at day 0 (median AUC = 5.707) but not meaningfully detectable in pups born to naïve dams (median AUC = 0.271) until day 7 (median AUC = 4.695) (Figure 4A). Anti-Spike IgG2a, the BALB/c-derived subclass present in maternal serum and transferable to pups, was detectable throughout the study; however, the anti-Spike IgG2a levels were much lower than the anti-Spike IgG1 levels (Figure 4B). Pups born to vaccinated dams showed modestly elevated IgG2a at day 0 (median AUC = 0.586) relative to pups born to naïve dams (median AUC = 0.141), consistent with maternally transferred anti-Spike IgG2a; levels converged between groups by day 21 (Figure 4B).

Notably, anti-Spike IgG2c, the exclusive readout of pup-derived de novo IgG production, was low and comparable between pups born to vaccinated and naïve dams across all time points, with a modest increase observed in both groups between days 0 (median AUC = 0.136 and 0.132) and 21 (median AUC = 0.142 and 0.198) (Figure 4C). The overall magnitude of anti-Spike IgG2c was low relative to total IgG1. These findings indicate that this vaccine elicited a primarily IgG1 response, ultimately limiting the ability to compare IgG2c between the two groups.

### 3.4. Neutralizing Antibody Responses to D614G SARS-CoV-2 Are Low and Heterogeneous Across Vaccination Groups

**Figure 5:**
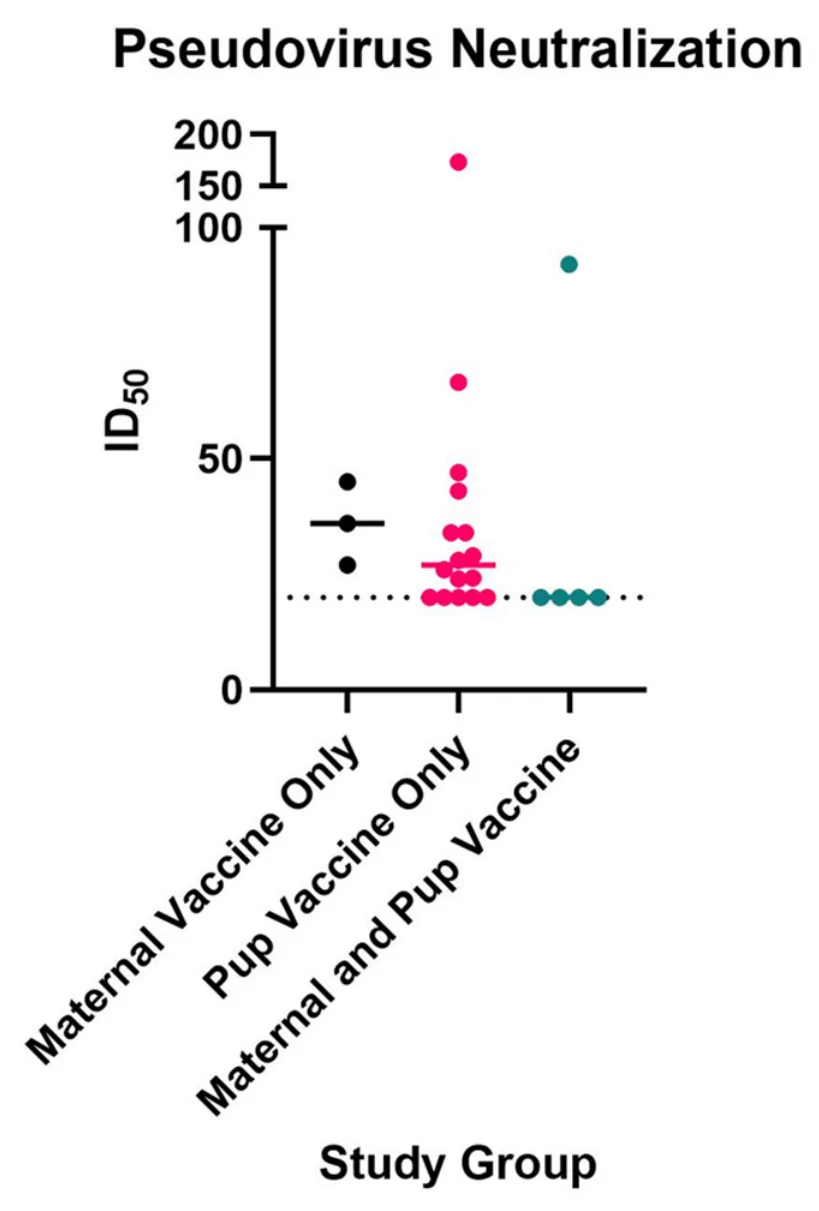
Pup neutralizing antibody responses to the SARS-CoV-2 pseudovirus on day 14 after vaccination. Neutralization titers (ID_50_) SARS-CoV-2 D614G strain pseudovirus (PSV) values are shown for sera collected from pups immunized at weaning. Serum was serially diluted in cell culture media and incubated for 45–90 min with pseudovirus expressing SARS-CoV-2 D614G Spike protein and a luciferase resporter enzyme. Cells were then added to each well, and plates were incubated for 72 h. Cell supernatant was then removed from each well, cells were lysed, lysates were incubated with Brite-Glo reagent and luminescence was measured. The dotted line represents the starting dilution of 1:20. Samples with an ID_50_ of <20 are set to 20.

To assess antibody function, we measured neutralizing activity against D614G SARS-CoV-2 pseudovirus in pup sera fourteen days post-pup immunization. Pups in all vaccine groups had either weak or undetectable neutralizing activity with few exceptions (Figure 5). Mock immunized pups exhibited a median ID_50_ (median ID_50_ = 36). In contrast, pups born to naïve dams showed the greatest inter-individual variability (ID_50_ min = 20, ID_50_ max = 173). Pups born to immunized dams demonstrated weak or undetectable neutralizing activity (ID_50_ min = 20, ID_50_ max = 92) with the exception of one animal with an elevated neutralizing titer (ID_50_ = 92), indicating that combined maternal and pup vaccination did not additively enhance pseudovirus neutralization. Across all groups, neutralizing activity was low and heterogeneous, with no vaccination group consistently achieving titers above the assay threshold, suggesting that the mRNA-LNP vaccine doses and schedule used in this study were insufficient to reliably elicit potent pseudovirus-neutralizing responses in early life regardless of maternal immunization status.

### 3.5. Early-Life mRNA-LNP Vaccination Induces Spike-Specific B Cells in the Presence of Maternal Antibody

**Figure 6:**
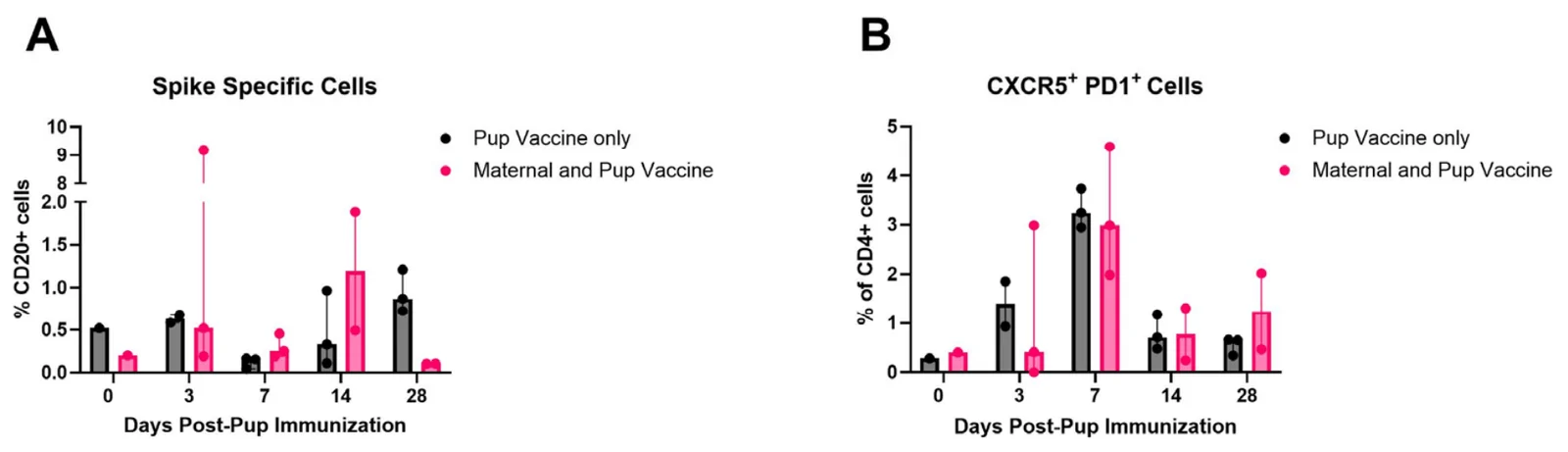
Splenic B cell and CD4+ T cell responses. B and T cell lymphocyte subsets were assessed in pups after SARS-CoV-2 Spike mRNA vaccination. (**A**) Spike-specific B cells; cells were stained with Spike protein tagged with either Alexafluor 488 or Brilliant violet 421. Data represents the proportion of CD20+ cells positive for both fluorophores. (**B**) CD4^+^CxC45^+^ PD1^+^ T follicular helper (TFh) cells. Representative gating strategies for all populations are shown in Supplementary Figure S1.

To characterize early cellular immune responses, splenic Spike-specific B cells and T follicular helper (Tfh) cells were assessed at days 0, 3, 7, 14, and 28 post-pup immunization in immunized weanling pups (Figure 6, Table 1). Spike-specific B cells were identified as CD20+ cells double-positive for SARS-CoV-2 Spike protein labeled with either Alexa Fluor 488– or Brilliant Violet 421 (Supplementary Figure S1).

Spike-specific B cell frequencies were detectable at low levels across all time points in both vaccination groups (Figure 6A). An elevation in Spike-specific B cells was observed at day 3 in pups born to immunized dams, with one animal exhibiting a markedly elevated response (~9% of CD20^+^ cells). Spike-specific B cell frequencies subsequently reduced in both groups through day 7, followed by a secondary increase at day 14 and then a decline toward baseline by day 28 in pups born to immunized dams but not in pups born to naïve dams. (Figure 6A).

CXCR5^+^PD1^+^ Tfh cell frequencies were low at days 0 and 3, increased substantially by day 7 in both vaccination groups, and then contracted through days 14 and 28 (Figure 6B). The broadly similar Tfh kinetics between vaccination groups indicate that pre-existing maternal antibody did not substantially alter the magnitude or timing of early Tfh responses following pup immunization. Together, these data demonstrate that early-life mRNA-LNP vaccination can elicit antigen-specific B cells and does not impact Tfh cell frequency within the spleen.

## 4. Discussion

Maternal antibodies provide essential early-life protection against infectious diseases, shielding newborns from severe outcomes associated with pathogens such as influenza, SARS-CoV-2, and respiratory syncytial virus [26,27,28,29]. Yet, maternal antibodies can inhibit newborn vaccine responses, and these interactions have shaped global vaccination schedules, often deferring vaccination until after maternal antibody levels decline, typically around 6 months of age [30]. Our findings provide evidence that this critical susceptibility gap in early infancy may be addressable via mRNA-LNP vaccines. Our work confirmed that this novel vaccine platform can elicit active humoral immunity in the presence of maternal antibody, raising the possibility of safely expanding vaccine protection into the earliest months of life.

We established that D614G Spike-specific maternal antibody was detectable in neonatal pups through at least 3 weeks post-pup immunization; however, neonatal pups did not develop robust humoral responses against SARS-CoV-2 Spike. This could indicate that the mouse neonatal period requires multiple doses to elicit robust vaccine-specific antibody kinetics at this age. This limitation warrants further studies to develop strongly immunogenic vaccines to facilitate modeling the effect of maternal antibody during this stage of development.

To assess how maternal immunity influences early humoral responses to mRNA-LNP vaccination, we examined the kinetics of anti-Spike IgG in weanling pups stratified by maternal immunization status. In pups born to immunized dams, anti-Spike IgG levels were elevated at baseline, reflecting passive maternal transfer, and remained stable through the study period, reflecting the concurrent waning of maternal antibody and accumulation of de novo pup-derived IgG. The steady decline in anti-Spike IgG in mock-immunized pups born to vaccinated dams over the monitoring period is consistent with known IgG half-life in mice and confirms that declining titers in the absence of active pup immunization reflects waning of passively acquired maternal antibody [22]. Importantly, at no time point after day 0 did anti-Spike IgG levels differ significantly between vaccinated pups born to naïve versus immunized dams, indicating that the magnitude and kinetics of de novo IgG accumulation was not substantially impaired by the presence of immunogen-specific maternal antibody. These findings differ from prior reports demonstrating marked maternal antibody interference with live-attenuated vaccines [8] and suggest that the mRNA-LNP platform may be less susceptible to this mechanism of immune suppression [18].

The crossbreed IgG subclass analysis provided a direct measure of pup-derived de novo antibody responses, leveraging strain-specific IgG subclass expression to unambiguously distinguish passively acquired maternal IgG from vaccine-elicited pup responses. IgG2c, expressed exclusively by C57BL/6-derived B cells, was detectable in hybrid pups irrespective of maternal immunization status; however, its overall magnitude was low relative to total IgG1, indicating that de novo pup-derived responses represented a minor fraction of the total antibody pool. This pattern likely reflects the Th2-skewed immune environment characteristic of BALB/c-background mice, in which IgG1 is the dominant subclass and IgG2a responses are inherently limited [24,25]. The absence of a significant difference in anti-Spike IgG2c between groups indicates that pre-existing maternal antibody did not suppress de novo pup B cell responses at the subclass level, though the sensitivity of IgG2c as a readout of vaccine-elicited immunity may be constrained in this hybrid model given the dominance of IgG1 in the total antibody pool.

Pseudovirus neutralization data revealed a notable dissociation between binding antibody titers and functional neutralizing activity. Despite robust IgG binding responses in vaccinated groups, neutralizing titers against D614G pseudovirus were low and variable across all groups. The highest median neutralization was observed in the pup-vaccine-only group. Most strikingly, pups that received both maternal and pup vaccination had the lowest neutralizing titers, suggesting that the mRNA-LNP SARS-CoV-2 vaccine doses used in this study did not efficiently elicit the potently neutralizing antibody responses seen in humans and in other murine studies. This functional dissociation between binding and neutralizing antibody in early life has been previously reported [14,31] and likely reflects qualitative differences in the specificity, avidity, and epitope targeting of early-life antibody responses, which may mature over time or with additional immunizations [14,32]. Factors including vaccine dose, formulation integrity, and immunization schedule may each contribute to the limited neutralizing activity observed here, highlighting the need for systematic evaluation of these parameters in preclinical early-life vaccination models. These findings highlight the need for prime-boost strategies in early-life vaccination and align with prior work in neonatal rhesus macaque models demonstrating that early-life HIV-1 Env-specific IgG responses can be durably boosted to improve antibody quality [17].

Early cellular immune responses, including antigen-specific B cell and Tfh cell expansion, were detectable in both vaccination groups and followed broadly similar kinetics, with Tfh cell frequencies peaking at day 7 post-immunization in both groups. These data demonstrate that the weanling mouse immune system can engage antigen-specific cellular pathways following mRNA-LNP vaccination even in the context of circulating maternal antibody. This is consistent with prior reports demonstrating that mRNA-1273 vaccination elicits Spike-specific CD4+ T cell responses and memory B cell responses in infants aged 6 months and older and that infant rhesus macaques vaccinated with mRNA-LNP SARS-CoV-2 vaccines develop durable S-specific memory B cells and T cell responses dominated by IFN-γ and TNF-α production [31,33]. Whether comparable cellular responses can be elicited in younger infants under 6 months of age, particularly in the presence of high levels of maternal antibody, remains an important open question that our murine model begins to address.

Several limitations should be considered when interpreting these findings. The initial study sought to compare relative level of responses between dam-immunized and unimmunized groups of pups and therefore was not designed to compare beyond-use-date formulations with freshly dispensed vaccine. The provided vaccine vials were used solely to enable timely conduct of experiments under conditions of vaccine scarcity. The immunological readouts were assessed at a limited number of time points and mice that had to be sacrificed to complete all assessments, and these data therefore represent snapshots of the immune response rather than a continuous longitudinal profile of individual animals. Third, neutralizing activity was measured against a single viral variant (D614G) at a single time point, limiting conclusions about the breadth or durability of functional antibody responses. IgG subclass analyses in crossbreed pups were confined to the early post-immunization period, and the durability of de novo pup IgG2c responses was not assessed. Finally, while this murine model outlines key features of human maternal antibody transfer, important species-specific differences exist, including the postnatal rather than transplacental route of IgG transfer in rodents and the non-equivalent functional mapping of mouse and human IgG subclasses [24,25], which should be considered when extrapolating these findings to human infants.

Together, our findings indicate that maternally derived antibodies shape the early kinetics and apparent magnitude of serological responses following early-life mRNA-LNP vaccination without preventing the initiation of de novo adaptive immunity. Although maternal immunity modulated the quantitative profile of early humoral responses, pups remained capable of mounting vaccine-elicited IgG responses and engaging antigen-specific B cell and Tfh cell pathways in the presence of passively acquired antibodies. The dissociation between antigen-binding and neutralizing activity, likely reflecting the qualitative immaturity of early-life humoral responses and the dosing constraints inherent to this study, shows the complexity of evaluating functional immunity in early life and the importance of optimizing vaccine formulation and schedule in preclinical models prior to clinical translation. This data suggests that mRNA-LNP vaccines at least partially overcome maternal antibody inhibition that can limit vaccine responses in early infancy; however, multiple doses are likely required for eliciting protective humoral responses in early life. Our findings do support the feasibility of extending mRNA-LNP vaccination into the earliest postnatal period and provide a preclinical framework for optimizing infant vaccine timing, dose, and schedule in the context of maternal immunity.

## Figures and Tables

**Table 1:** Experimental vaccine groups.

Group	Maternal Vaccine (mcg)	Pup Vaccine (mcg)	Pup Serum (n)	Pup Spleen (n)
Neonatal Studies				
Maternal and Pup Immunization	5	4	Total = 13	Total = 13
			14 dpi (5)	14 dpi (5)
			21 dpi (4)	21 dpi (4)
Pup Immunization	0	4	Total = 13	Total = 13
			14 dpi (5)	14 dpi (5)
			21 dpi (6)	21 dpi (6)
Weanling Studies				
Maternal Immunization only	5	0	Total = 18	Total = 22
			3 dpi (4)	3 dpi (4)
			7 dpi (2)	7 dpi (4)
			14 dpi (5)	14 dpi (5)
			21 dpi (0)	21 dpi (2)
			28 dpi (2)	28 dpi (2)
			35 dpi (5)	35 dpi (5)
Maternal and Pup Immunization	5	5	Total = 100	Total = 25
			0 dpi (29)	0 dpi (3)
			3 dpi (25)	3 dpi (4)
			7 dpi (18)	7 dpi (5)
			14 dpi (16)	14 dpi (5)
			21 dpi (0)	21 dpi (0)
			28 dpi (7)	28 dpi (4)
			35 dpi (5)	35 dpi (3)
Pup Immunization	5	5	Total = 63	Total = 25
			0 dpi (4)	0 dpi (3)
			3 dpi (3)	3 dpi (4)
			7 dpi (13)	7 dpi (5)
			14 dpi (26)	14 dpi (6)
			21 dpi (3)	21 dpi (0)
			28 dpi (9)	28 dpi (4)
			35 dpi (5)	35 dpi (3)
Crossbreed Studies				
Maternal and Pup Immunization	5	5	Total = 9	Total = 9
			0 dpi (2)	0 dpi (2)
			7 dpi (8)	7 dpi (8)
			21 dpi (3)	21 dpi (3)
Pup Immunization	0	5	Total = 8	Total = 8
			0 dpi (1)	0 dpi (1)
			7 dpi (3)	7 dpi (3)
			21 dpi (3)	21 dpi (3)

**Table 2:** Flow cytometry panel—antibodies and reagents.

#	Marker	Fluorochrome	Clone
1	CD20	Alexa Fluor 350	SA275A11
2	CXCR5	BB700	2G8
3	CD3	PE-eFluor 610	17A2
4	CD4	Alexa Fluor 700	RM4-5
5	Spike (SARS-CoV-2)	Streptavidin-AF488	N/A (biotinylated protein)
6	PD1	Super Bright 780	J43
7	Spike (SARS-CoV-2)	Streptavidin-BV421	N/A (biotinylated protein)
8	Viability	LIVE/DEAD Lime (506)	N/A
9	Ki67	Alexa Fluor 647	B56/ab155800
10	BCL6	PE	7D1

## Data Availability

Data supporting the reported results are available from the corresponding author upon reasonable request.

## Supplementary Materials

The following supporting information can be downloaded at: https://www.mdpi.com/article/10.3390/vaccines14090769/s1, Figure S1: Flow Cytometry Gating Strategy.

## References

[1] Burns M.D., Muir C., Atyeo C., Davis J.P., Demidkin S., Akinwunmi B., Fasano A., Gray K.J., Alter G., Shook L.L. (2022). Relationship between Anti-Spike Antibodies and Risk of SARS-CoV-2 Infection in Infants Born to COVID-19 Vaccinated Mothers. Vaccines.

[2] Henkle E., Steinhoff M.C., Omer S.B., Roy E., Arifeen S.E., Raqib R., Breiman R.F., Caulfield L.E., Moss W.J., Zaman K. (2013). The effect of exclusive breast-feeding on respiratory illness in young infants in a maternal immunization trial in Bangladesh. Pediatr. Infect. Dis. J..

[3] Langel S.N., Blasi M., Permar S.R. (2022). Maternal immune protection against infectious diseases. Cell Host Microbe.

[4] Jiang Y., Patel C.D., Manivanh R., North B., Backes I.M., Posner D.A., Gilli F., Pachner A.R., Nguyen L.N., Leib D.A. (2017). Maternal Antiviral Immunoglobulin Accumulates in Neural Tissue of Neonates To Prevent HSV Neurological Disease. mBio.

[5] Karrow N.A., Shandilya U.K., Pelech S., Wagter-Lesperance L., McLeod D., Bridle B., Mallard B.A. (2021). Maternal COVID-19 Vaccination and Its Potential Impact on Fetal and Neonatal Development. Vaccines.

[6] Kim D., Huey D., Oglesbee M., Niewiesk S. (2011). Insights into the regulatory mechanism controlling the inhibition of vaccine-induced seroconversion by maternal antibodies. Blood.

[7] van der Staak M., Ten Hulscher H.I., Nicolaie A.M., Smits G.P., de Swart R.L., de Wit J., Rots N.Y., van Binnendijk R.S. (2025). Long-term Dynamics of Measles Virus-Specific Neutralizing Antibodies in Children Vaccinated Before 12 Months of Age. Clin. Infect. Dis..

[8] Mok D.Z.L., Chan K.R. (2020). The Effects of Pre-Existing Antibodies on Live-Attenuated Viral Vaccines. Viruses.

[9] Siegrist C.A. (2003). Mechanisms by which maternal antibodies influence infant vaccine responses: Review of hypotheses and definition of main determinants. Vaccine.

[10] Wanlapakorn N., Maertens K., Vongpunsawad S., Puenpa J., Tran T.M.P., Hens N., Van Damme P., Thiriard A., Raze D., Locht C. (2020). Quantity and Quality of Antibodies After Acellular Versus Whole-cell Pertussis Vaccines in Infants Born to Mothers Who Received Tetanus, Diphtheria, and Acellular Pertussis Vaccine During Pregnancy: A Randomized Trial. Clin. Infect. Dis..

[11] Panagiotakopoulos L., Moulia D.L., Godfrey M., Link-Gelles R., Roper L., Havers F.P., Taylor C.A., Stokley S., Talbot H.K., Schechter R. (2024). Use of COVID-19 Vaccines for Persons Aged >/=6 Months: Recommendations of the Advisory Committee on Immunization Practices—United States, 2024—2025. MMWR. Morb. Mortal. Wkly. Rep..

[12] Munoz F.M., Sher L.D., Sabharwal C., Gurtman A., Xu X., Kitchin N., Lockhart S., Riesenberg R., Sexter J.M., Czajka H. (2023). Evaluation of BNT162b2 COVID-19 Vaccine in Children Younger than 5 Years of Age. N. Engl. J. Med..

[13] Anderson E.J., Creech C.B., Berthaud V., Piramzadian A., Johnson K.A., Zervos M., Garner F., Griffin C., Palanpurwala K., Turner M. (2022). Evaluation of mRNA-1273 Vaccine in Children 6 Months to 5 Years of Age. N. Engl. J. Med..

[14] Dalapati T., Williams C.A., Giorgi E.E., Hurst J.H., Herbek S., Chen J.L., Kosman C., Rotta A.T., Turner N.A., Pulido N. (2024). Immunogenicity of Monovalent mRNA-1273 and BNT162b2 Vaccines in Children <5 Years of Age. Pediatrics.

[15] Otero C.E., Langel S.N., Blasi M., Permar S.R. (2020). Maternal antibody interference contributes to reduced rotavirus vaccine efficacy in developing countries. PLoS Pathog..

[16] Niewiesk S. (2014). Maternal antibodies: Clinical significance, mechanism of interference with immune responses, and possible vaccination strategies. Front. Immunol..

[17] Curtis A.D., Dennis M., Eudailey J., Walter K.L., Cronin K., Alam S.M., Choudhary N., Tuck R.H., Hudgens M., Kozlowski P.A. (2020). HIV Env-Specific IgG Antibodies Induced by Vaccination of Neonatal Rhesus Macaques Persist and Can Be Augmented by a Late Booster Immunization in Infancy. mSphere.

[18] LaTourette P.C., Awasthi S., Desmond A., Pardi N., Cohen G.H., Weissman D., Friedman H.M. (2020). Protection against herpes simplex virus type 2 infection in a neonatal murine model using a trivalent nucleoside-modified mRNA in lipid nanoparticle vaccine. Vaccine.

[19] Willis E., Pardi N., Parkhouse K., Mui B.L., Tam Y.K., Weissman D., Hensley S.E. (2020). Nucleoside-modified mRNA vaccination partially overcomes maternal antibody inhibition of de novo immune responses in mice. Sci. Transl. Med..

[20] Yoshida M., Claypool S.M., Wagner J.S., Mizoguchi E., Mizoguchi A., Roopenian D.C., Lencer W.I., Blumberg R.S. (2004). Human neonatal Fc receptor mediates transport of IgG into luminal secretions for delivery of antigens to mucosal dendritic cells. Immunity.

[21] Pyzik M., Rath T., Lencer W.I., Baker K., Blumberg R.S. (2015). FcRn: The Architect Behind the Immune and Nonimmune Functions of IgG and Albumin. J. Immunol..

[22] Ober R.J., Martinez C., Vaccaro C., Zhou J., Ward E.S. (2004). Visualizing the site and dynamics of IgG salvage by the MHC class I-related receptor, FcRn. J. Immunol..

[23] Williams C.A., Wong T.A.S., Ball A.H., Lieberman M.M., Lehrer A.T. (2022). Maternal Immunization Using a Protein Subunit Vaccine Mediates Passive Immunity against Zaire ebolavirus in a Murine Model. Viruses.

[24] Nazeri S., Zakeri S., Mehrizi A.A., Sardari S., Djadid N.D. (2020). Measuring of IgG2c isotype instead of IgG2a in immunized C57BL/6 mice with Plasmodium vivax TRAP as a subunit vaccine candidate in order to correct interpretation of Th1 versus Th2 immune response. Exp. Parasitol..

[25] Martin R.M., Brady J.L., Lew A.M. (1998). The need for IgG2c specific antiserum when isotyping antibodies from C57BL/6 and NOD mice. J. Immunol. Methods.

[26] Nazir Z., Habib A., Ali T., Singh A., Zulfiqar E., Haque M.A. (2024). Milestone in infant health: Unveiling the RSV vaccine’s shielding effect for newborns. Int. J. Surg..

[27] Cinicola B., Conti M.G., Terrin G., Sgrulletti M., Elfeky R., Carsetti R., Fernandez Salinas A., Piano Mortari E., Brindisi G., De Curtis M. (2021). The Protective Role of Maternal Immunization in Early Life. Front. Pediatr..

[28] Albrecht M., Arck P.C. (2020). Vertically Transferred Immunity in Neonates: Mothers, Mechanisms and Mediators. Front. Immunol..

[29] Patel C.D., Backes I.M., Taylor S.A., Jiang Y., Marchant A., Pesola J.M., Coen D.M., Knipe D.M., Ackerman M.E., Leib D.A. (2019). Maternal immunization confers protection against neonatal herpes simplex mortality and behavioral morbidity. Sci. Transl. Med..

[30] Semmes E.C., Chen J.L., Goswami R., Burt T.D., Permar S.R., Fouda G.G. (2020). Understanding Early-Life Adaptive Immunity to Guide Interventions for Pediatric Health. Front. Immunol..

[31] Garrido C., Curtis A.D., Dennis M., Pathak S.H., Gao H., Montefiori D., Tomai M., Fox C.B., Kozlowski P.A., Scobey T. (2021). SARS-CoV-2 vaccines elicit durable immune responses in infant rhesus macaques. Sci. Immunol..

[32] Kollmann T.R., Levy O., Montgomery R.R., Goriely S. (2012). Innate immune function by Toll-like receptors: Distinct responses in newborns and the elderly. Immunity.

[33] Rostad C.A., Campbell J.D., Paulsen G.C., Ghamloush S.S., Xu W., Zheng L., McElrath M.J., De Rosa S.C., Girard B., Das R. (2025). Evaluation of Cellular Immune Responses After mRNA-1273 Vaccination in Children 6 Months to 11 Years of Age. J. Infect. Dis..

